# Instilling eye drops and ointment in a baby or young child

**Published:** 2010-03

**Authors:** Ingrid Mason, Sue Stevens

**Affiliations:** CBM Capacity Development Officer and Medical Advisor, PO Box 58004, 00200 City Square, Ring Road Parklands, Nairobi, Kenya.; Former Nurse Advisor, *Community Eye Health Journal,* International Centre for Eye Health, London School of Hygiene and Tropical Medicine, Keppel Street, London WC1E 7HT, UK.

**Figure:**
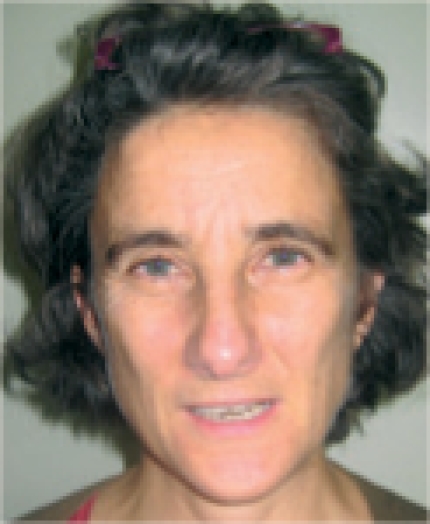


**Figure:**
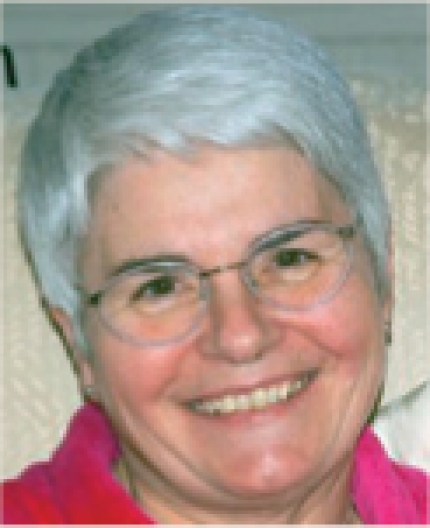


Extra care is necessary when instilling eye drops and eye ointment in babies and children. It is also important that parents and carers are taught how to continue the treatment when the child leaves the eye clinic or hospital setting. Allow parents to practice, and supervise and support them until they feel confident to do it on their own.

## Before performing this procedure

- Wash your hands (and afterwards too).
- Ask the parent and any other helper to wash their hands.
- Minimise distractions.
- Ensure good lighting.
- Explain to the parent (and child, if old enough to understand) that the medicine needs to be put into the eye and that it will make the eye better.
- Explain that, once the medication has been put in, the vision may be blurred for some time.
- Some eye drops cause a stinging sensation - tell the parent and the child so they can expect it: doing so is important as it builds trust.

## You will need

- Eye drops
- Ointment
- Treatment card or prescription note
- Cotton wool, swab, or paper tissue
- Cooled boiled water, if the eye needs cleaning
- A toy or colourful picture

## Preparation

- Show the child what the container of eye drops/ointment looks like. Put some on the back of the child's hand so he or she knows what it feels like.
- Use your finger to point at your own eye and show where the drop/ointment is going to be instilled. You can also pretend to instil some in the parent's eye to show the child what to expect.
- Ask the parent to hold the child in a gentle, comforting manner.
- Encourage the parent to speak to the child in a comforting way throughout the procedure and to cuddle the child immediately afterwards.
- A baby or child who is too young to cooperate may be wrapped in a sheet or blanket to restrain their arms (see Figure 8 on page 6).
- Work as quickly and calmly as you can - this minimises the child's distress.

## Method

- Check the medication label against the treatment chart (if in hospital) or against the prescription note (if at home).
- Ensure the eye(s) are clean. To clean the eye, moisten cotton wool, a swab, or a paper tissue with cooled, boiled water and gently wipe the closed eye **from the inner to outer** canthus. Use **each** swab/cotton wool/tissue **once only.**
- Ask the child to look upwards. You can ask a helper to hold up a toy. Alternatively, attach a toy or colourful picture to the ceiling.
- Gently pull down the lower eyelid to create a ‘sac’ (Figure 1).

**Figure:**
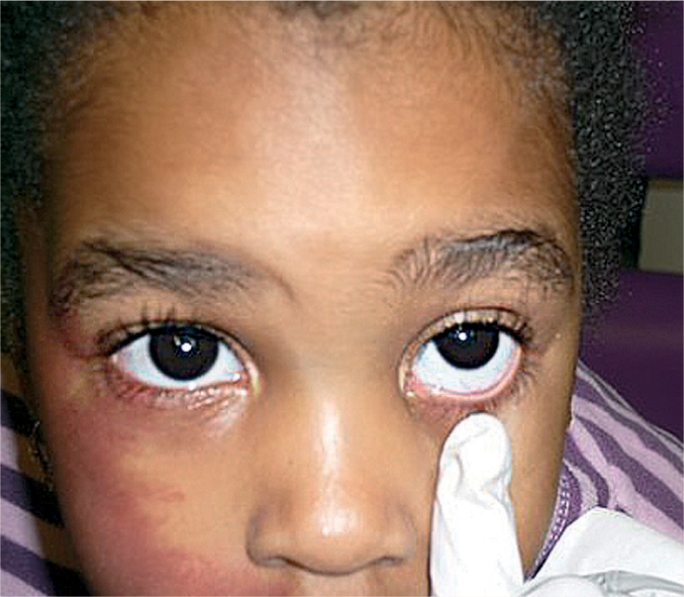
Figure 1

Bronwen Walters, Great Ormond Street Children's Hospital

NOTE: In the case of a child who is unable to cooperate, you will need to ease the eye open gently **(do not pull),** by holding the upper and lower eyelids apart simultaneously while you instil the drop or ointment (Figure 2). It is very important to avoid any pressure on the eye.

**Figure:**
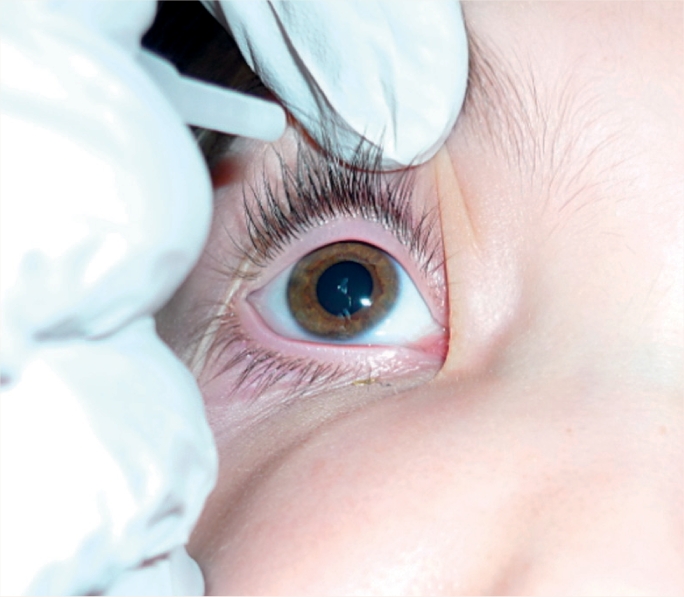
Figure 2

Bronwen Walters, Great Ormond Street Children's Hospital

- Hold the tip of the bottle, dropper, or tube close to the eye, but do not allow it to touch the eye. Instil **one** drop (Figure 3) or apply a line of ointment (Figure 4) in the ‘sac’. Release the eyelid(s) so the eye can close.

**Figure:**
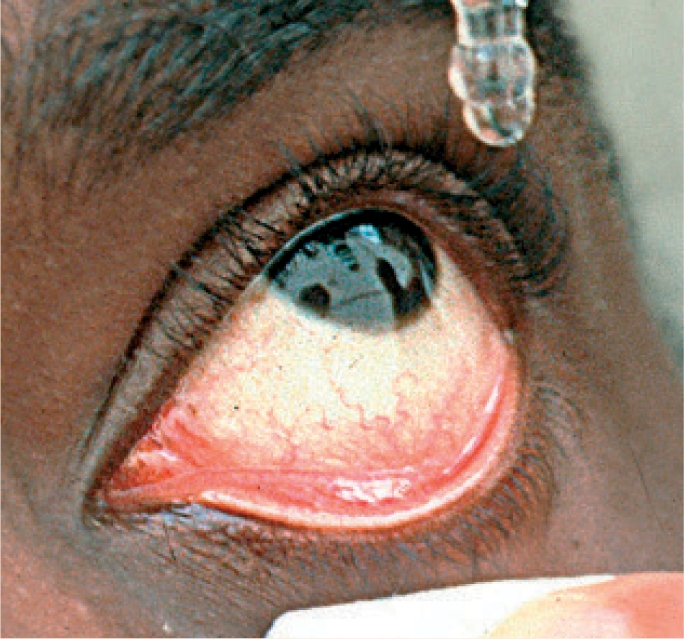
Figure 3

Pak Sang Lee

**Figure:**
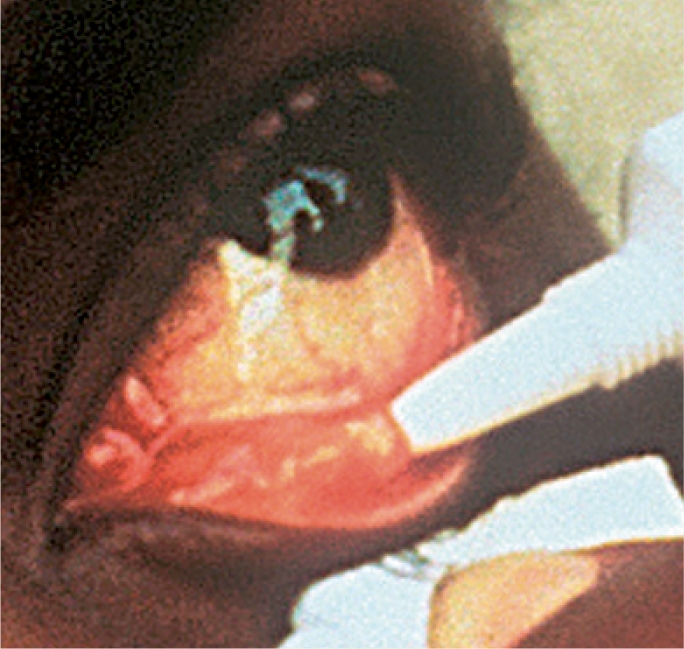
Figure 4

ICEH

Wait three to five minutes before instilling any other prescribed drop or ointment.

## After the procedure

- Replace the top of the bottle, dropper, or tube immediately after use.
- Reassure the parent and the child that you have finished.
- Wash your hands.
- Praise the child and emphasise how well the child has behaved - whatever the reaction has been!
- With agreement from the parent, give the child some sort of reward, such as a sweet or a special food or toy. For babies, encourage the mother to put them to her breast.
- If the child has an infection in either eye, ensure that the bottle or tube is only used for this child and thrown away when the child is discharged, or given only to this child to take home.

## Advising parents who have to continue treatment at home

Advise the parent to ask someone to help them hold the child when instilling the eye drops or ointment. If this is not possible, they can wrap the child in a blanket so their arms are restrained. A helper may still be needed to gently hold the eye open as described above. Ensure that any helpers have washed their hands.

